# The use of magnesium sulfate can reduce the mortality risk of cirrhosis patients: a retrospective cohort study

**DOI:** 10.3389/fphar.2025.1551495

**Published:** 2025-10-20

**Authors:** Boxian Chen, Yuping Yang, Mouji Liang, Yanqi Kou, Ruyin Ye, Liping Zhan, Yujie Huang, Qing Zhang, Haoyuan Huang, Jieming Zheng, Zhe Huang, Shicai Ye

**Affiliations:** ^1^ Department of Gastroenterology, Affiliated Hospital of Guangdong Medical University, Guangdong Medical University, Zhanjiang, Guangdong, China; ^2^ Department of Colorectal Surgery, Affiliated Hospital of Guangdong Medical University, Guangdong Medical University, Zhanjiang, Guangdong, China

**Keywords:** liver cirrhosis, magnesium sulfate, MIMIC-IV, all-cause mortality, propensity score matching

## Abstract

**Background:**

Magnesium deficiency is common in patients with cirrhosis, but there is a lack of real-world evidence to support the effect of magnesium supplementation on prognosis.

**Objective:**

To explore whether magnesium sulfate supplementation is beneficial for patients with cirrhosis using data from the MIMIC-IV database.

**Methods:**

Patients with cirrhosis were divided into magnesium sulfate group and non-magnesium sulfate group according to medication use during hospitalization after admission to the intensive care unit (ICU). In-hospital all-cause mortality was the primary outcome, and 180-day all-cause mortality was the secondary outcome. Propensity score matching (PSM) method, univariate and multivariate regression analysis were used to evaluate the effect of magnesium sulfate on prognosis, and Kaplan-Meier curves, subgroup analysis and sensitivity analysis were performed to clarify the stability of the results.

**Results:**

The prematched cohort included 3,312 patients, while the propensity score matched cohort included 1,308 patients. In the PSM analysis, the in-hospital all-cause mortality in the magnesium sulfate group was 22.0% (144/654), and that in the non-use group was 31.0% (203/654). Magnesium sulfate use was associated with lower in-hospital mortality (odds ratio [OR], 0.47; 95% confidence interval [CI], 0.33–0.69; P < 0.001) and reduced all-cause mortality at 180 days after ICU admission (hazard ratio [HR], 0.61; 95% CI, 0.51–0.72; P < 0.001). Sensitivity analyses confirmed the robustness of these results.

**Conclusion:**

Magnesium sulfate use is associated with reduced in-hospital and 180-day all-cause mortality in ICU patients with cirrhosis, which needs to be verified in prospective studies.

## 1 Introduction

Cirrhosis is a terminal disease of various chronic liver diseases, characterized by chronic inflammation, diffuse fibrosis, pseudolobular formation, and portal-systemic circulation in the liver (Parola and Pinzani, 2019). The most common causes include viral hepatitis, alcoholic liver disease, and metabolic fatty liver disease (Paducheva, 2023). As of 2017, there were approximately 10.6 million patients with decompensated cirrhosis and 112 million patients with compensated cirrhosis worldwide, resulting in approximately 1.16 million deaths each year, ranking 11th in the world (Asrani et al., 2019; Sepanlou et al., 2020). Effective clinical interventions are crucial to reduce the risk of death in patients with cirrhosis.

Magnesium ion is the second most abundant cation in human cells and is considered a cofactor for many enzymatic reactions (Van Laecke, 2019). More than 99% of the total Mg^2^+ in the body is located within cells and is mainly stored in bones (50%–65%), where it participates in the formation of bones together with calcium and phosphorus, and also participates in the formation of muscles, soft tissues and organs (34%–39%), while less than 1%–2% of Mg^2^+ exists in blood and extracellular fluid (Konrad et al., 2004; Schuchardt and Hahn, 2017). Serum magnesium levels do not usually reflect magnesium levels in different parts of the body because, even when magnesium intake is reduced and magnesium is deficient, magnesium can still be obtained from bones (as well as muscles and internal organs) to maintain normal serum magnesium levels (Rude and Gruber, 2004). Therefore, although serum values are within normal range, the body may be in a state of severe Mg 2+ depletion (De Baaij et al., 2015; DiNicolantonio et al., 2018). Only after a long-term magnesium deficiency can patients develop clinically relevant hypomagnesemia. Most studies conducted in humans have found that blood magnesium concentrations in patients with cirrhosis are lower than those in healthy controls (Rocchi et al., 1994; Koivisto et al., 2002; Kar et al., 2014; Nangliya et al., 2015; Cohen-Hagai et al., 2018; Peng et al., 2021).

In addition, studies have found that magnesium deficiency in hepatocytes and overexpression of the endogenous enzyme TRPM7 are associated with the severity of hepatocyte damage and prognosis in patients with cirrhosis by using atomic absorption spectroscopy and synchrotron X-ray fluorescence microscopy (Parisse et al., 2023). Magnesium ions are essential for ATP metabolism, DNA and RNA synthesis, reproduction, and protein synthesis. Can magnesium supplementation improve outcomes in patients with cirrhosis? Clinical research on this topic is limited.

Magnesium sulfate is a commonly used therapeutic drug. Its main indications include pre-eclampsia and eclampsia, arrhythmias and perioperative pain, and correction of hypomagnesemia (Kunst et al., 2019; Shin et al., 2020; Scott et al., 2022). In addition, studies have shown that magnesium sulfate can restore sinus rhythm in critically ill patients with new-onset atrial fibrillation (Johnston et al., 2022).

There are still limited studies on the specific application of magnesium sulfate in patients with cirrhosis and its effect on mortality. This study aims to explore the relationship between the application of magnesium sulfate and mortality in patients with cirrhosis through a retrospective cohort study, in order to provide further evidence for clinical practice and provide new perspectives for potential treatment strategies.

## 2 Materials and methods

### 2.1 Database introduction

This study is a retrospective cohort study in which all data were obtained from the Medical Information Mart for Intensive Care IV (MIMIC-IV) database. The MIMIC-IV database is an extended, freely accessible resource and an important asset for the global research community focused on critical care (Johnson et al., 2023). It was developed by the Massachusetts Institute of Technology (MIT) in collaboration with Beth Israel Deaconess Medical Center and contains detailed health data from more than 40,000 patients hospitalized between 2008 and 2019. The database includes a wide range of information such as demographic details, vital signs, lab test results, medications, and diagnostic codes, providing a comprehensive view of ICU patient care. The first author, Boxian Chen, who has completed the Collaborative Institutional Training Initiative (CITI) program and passed the “Conflicts of Interest” and “Data or Specimens Only Research” examinations (Certification ID: 63562939), was authorized to access the MIMIC-IV database. Patient privacy was safeguarded through the use of anonymous personal identifiers, eliminating the need for informed consent.

### 2.2 Population selection criteria

**FIGURE 1:**
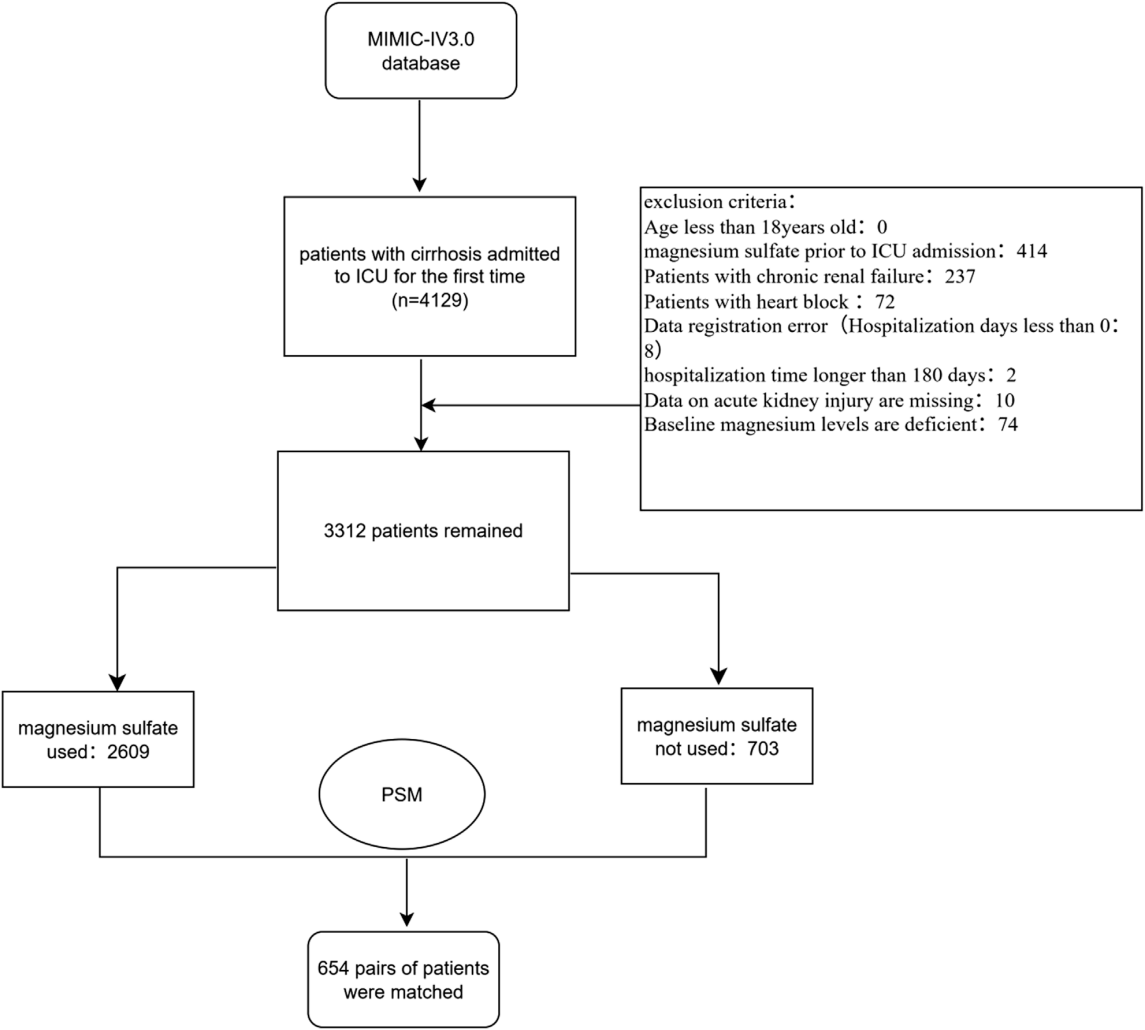
Flowchart of the study. Abbreviation: PSM, Propensity score matching; MIMIC-IV, Medical Information Mart for Intensive Care IV.

Patients whose diagnosis included “cirrhosis” and who were admitted to the ICU for the first time were included in the study. We collected 4,129 hospitalization records of patients with cirrhosis from the MIMIC-IV database. Exclusion criteria are as follows:1. Patients under 18 years old; 2. Patients who used magnesium sulfate before admission to the ICU; 3. Patients with chronic renal failure or heart block; 4. Patients with incorrect data registration (hospitalization time less than 0); 5. Patients with hospitalization time more than 180 days; 6. Patients with missing data on acute kidney injury; 7. Patients with missing baseline blood magnesium concentration. The ICD codes for chronic renal failure and heart block are shown in Supplementary Material S14, S15, respectively. We defined the first blood magnesium concentration on the first day after the patient was admitted to the ICU as the baseline blood magnesium concentration. Ultimately, we identified 3,312 patients with cirrhosis during their first ICU admission. The ICD versions, codes, and diagnostic names of the patients included in the study are shown in Supplementary Material S1. The detailed screening process for the entire study cohort is illustrated in Figure 1. The study cohort was divided into two groups: those who received magnesium sulfate treatment (magnesium sulfate use group) and those who did not (no use group).

### 2.3 Magnesium sulfate exposure

The exposure factor was whether intravenous magnesium sulfate was used after ICU admission, without any restrictions. The information on magnesium sulfate use was obtained from the prescriptions table. Patients with missing data regarding magnesium sulfate exposure were excluded from the analysis.

### 2.4 Data extraction

Data extraction was performed using Structured Query Language (SQL). The SQL script code was obtained from the GitHub repository (https://github.com/MIT-LCP/mimic-iv). Basic characteristics of the patients were collected, including age, gender, race, weight, and height. We extracted treatment data include invasive ventilation and continuous renal replacement therapy (CRRT). We also extracted information on comorbidities and complications according to the International Classification of Diseases coding system, including myocardial infarction, congestive heart failure, peripheral vascular disease, chronic pulmonary disease, diabetes, renal disease, cancer, hypertension, sepsis, ascites, hepatopulmonary syndrome (HPS), hepatorenal syndrome (HS), hepatic encephalopathy (HE), portal hypertension (PH), diarrhea, spontaneous bacterial peritonitis (SBP), acute kidney injury (AKI). We collected data from the first laboratory tests after admission to the ICU, including red blood cell (RBC), white blood cell (WBC), platelets, hemoglobin, magnesium, potassium, sodium, calcium, chloride, bicarbonate, creatinine, blood urea nitrogen (BUN), aniongap, prothrombin time (PT), activated partial thromboplastin time (APTT), alanine aminotransferase (ALT), aspartate aminotransferase (AST), alkaline phosphatase (ALP), total bilirubin. We also recorded the first disease severity scores (Simplified Acute Physiology ScoreII(SAPSII), charlson comorbidity index (CCI), glasgow coma scale (GCS), Model for End-Stage Liver Disease (MELD), oxford acute severity of illness score (OASIS) and sequential organ failure assessment (SOFA)), and mean vital signs (temperature, respiratory rate (RR), heart rate (HR), mean blood pressure (MBP), oxygen saturation (Spo2)) for the first day after admission to the ICU.

### 2.5 Primary and secondary outcomes

The primary outcome was in-hospital all-cause mortality. Secondary outcomes included all-cause mortality within 180 days after admission to the ICU.

### 2.6 Propensity score matching (PSM)

We used propensity score matching to adjust for variables (Yao et al., 2017). The probability of each patient receiving magnesium sulfate (i.e., propensity score) was obtained through logistic regression modeling. Variables included in the propensity score model for matching were demographic characteristics (age, gender), presence of underlying diseases (myocardial infarction, congestive heart failure, peripheral vascular disease, chronic lung disease, diabetes mellitus, cancer, hypertension), presence of comorbidities (ascites, HE, HS, PH, SBP, HPS, sepsis, AKI), vital signs (HR, MBP, RR, temperature, Spo2), disease severity scores (SOFA score, SAPS II score, Charlson Comorbidity Index, MELD score), laboratory tests (RBC, WBC, platelets, creatinine, magnesium, calcium, chloride, bicarbonate potassium, sodium, BUN), and treatment presence (invasive ventilation, CRRT) were also included. Matching was performed using the nearest neighbor method at a 1:1 ratio, with a caliper width of 0.1, without replacement. The balance of variables between groups before and after matching was assessed using standardized mean deviation (SMD), with SMD values less than 0.10 indicating a balanced distribution. The primary analyses were conducted in the matched cohort and aimed to examine the association between magnesium sulfate use and primary and secondary outcomes. The distribution of propensity scores before and after matching is shown in Supplementary Material S2.

### 2.7 Statistical analysis

As a retrospective analysis, the sample size was based on the data available in the database. Missing rates for each variable are shown in Supplementary Material S3. We excluded variables with more than 25% missing data. For variables with a missing rate of less than 25%, multiple imputation was used to estimate missing values, assuming that the data were missing at random (He, 2010). Normality tests indicated that all continuous variables in this study did not conform to a normal distribution, and therefore, they are presented as medians and interquartile ranges. Comparisons between groups were made using the chi-square test or Fisher‘s exact test for categorical variables, and the Mann-Whitney U test for continuous variables. Standardized mean difference (SMD) was used to represent differences in variables between groups in the original and matched cohorts. To assess the effect of magnesium sulfate use on survival prognosis, multifactorial logistic regression models were created to generate odds ratio (OR) for the primary outcome and their 95% confidence interval (CI). Cox proportional hazards models were created to generate hazard ratio (HR) and 95% CIs for the secondary outcome to determine the independent effect of magnesium sulfate use on patient outcomes. Multivariance expansion factor (VIF) was used to detect multicollinearity between the included variables before performing multivariate logistics regression. A VIF of less than 5 for each variable indicates the absence of multicollinearity (See Supplementary Material S4). The cumulative incidence of all-cause mortality over 180 days was analyzed using the Kaplan-Meier method and assessed using the log-rank test. In addition, we conducted subgroup analyses based on the following factors: age (<60 vs. ≥ 60 years), sex, diabetes, renal disease, hypertension, sepsis, ascites, CRRT, HS, HE, PH, diarrhea, SBP. A two-tailed P < 0.05 was considered statistically significant in all analyses. Statistical analyses were performed using R software (version 4.2.3).

### 2.8 Sensitivity analyses

We conducted three sensitivity analyses. First, we performed sensitivity analyses on the entire dataset. Then, recognizing that magnesium sulfate may be prognostically beneficial for patients with sepsis (Gu et al., 2023), we conducted sensitivity analyses again by excluding patients with sepsis from the entire dataset. Finally, since some patients took oral magnesium ion preparations (including magnesium oxide, magnesium hydroxide, and magnesium citrate), we excluded these patients and conducted a sensitivity analysis again. With these three sensitivity analyses, we assessed the robustness of the findings obtained from the matched cohort.

## 3 Results

### 3.1 Patient characteristics

Before PSM, there were significant differences between the two groups in terms of age, gender, disease severity score, underlying diseases, and complications. After PSM, all variables included in PSM were well balanced between the two groups (SMD <0.10). The baseline characteristics of the unmatched cohort and the matched cohort are shown in Table 1. The distribution balance before and after propensity score matching is shown in Supplementary Material S2.

**TABLE 1:** Baseline characteristics between two groups before PSM and after PSM.

Variable	Before PSM					After PSM				
	Total (n = 3,312)	No magnesium sulfate (n = 703)	Magnesium sulfate (n = 2,609)	*P*	SMD	Total (n = 1,352)	No magnesium sulfate (n = 676)	Magnesium sulfate (n = 676)	*P*	SMD
Age, Years	60.11 (52.46, 68.05)	60.49 (53.59, 68.78)	59.98 (52.11, 67.87)	0.034	0.101	61.10 (53.61, 68.78)	60.48 (53.57, 68.36)	61.82 (53.87, 69.25)	0.281	0.036
RBC, m/μL	3.07 (2.60, 3.62)	3.08 (2.60, 3.62)	3.06 (2.60, 3.61)	0.446	0.042	3.07 (2.59, 3.62)	3.08 (2.59, 3.63)	3.06 (2.60, 3.60)	0.761	0.02
WBC, K/uL	9.10 (5.80, 14.30)	9.10 (5.70, 14.00)	9.10 (5.90, 14.40)	0.872	0.048	9.10 (5.80, 13.83)	9.05 (5.60, 13.88)	9.20 (6.03, 13.78)	0.636	0.066
Platelet, K/uL	107.00 (68.00, 162.00)	113.00 (72.00, 171.00)	105.00 (67.00, 159.00)	0.045	0.061	111.50 (72.00, 171.00)	113.00 (72.00, 172.00)	110.00 (71.25, 168.75)	0.929	0.031
Hemoglobin, g/dL	9.60 (8.20, 11.20)	9.60 (8.30, 11.40)	9.60 (8.20, 11.20)	0.137	0.077	9.60 (8.20, 11.30)	9.60 (8.30, 11.50)	9.60 (8.12, 11.20)	0.383	0.061
PT,s	17.70 (14.70, 22.50)	17.80 (14.60, 23.90)	17.70 (14.70, 22.20)	0.134	0.111	17.90 (14.60, 23.13)	17.60 (14.53, 23.35)	18.20 (14.70, 22.80)	0.857	0.023
PTT,s	35.90 (30.67, 45.00)	36.70 (31.30, 47.55)	35.70 (30.60, 44.10)	0.002	0.095	36.70 (31.17, 46.62)	36.55 (31.10, 46.50)	37.05 (31.20, 46.70)	0.74	0.038
Sodium, mEq/L	137.00 (133.00, 140.00)	136.00 (132.00, 140.00)	137.00 (134.00, 141.00)	<0.001	0.205	136.00 (132.00, 140.00)	136.00 (132.00, 140.00)	136.00 (132.00, 140.00)	0.958	0.011
Creatinine, mg/dL	1.10 (0.80, 1.80)	1.30 (0.90, 2.40)	1.00 (0.70, 1.70)	<0.001	0.364	1.30 (0.80, 2.10)	1.30 (0.80, 2.20)	1.30 (0.80, 2.10)	0.324	0.009
Aniongap, mmol/L	14.00 (12.00, 18.00)	14.00 (12.00, 19.00)	14.00 (12.00, 18.00)	0.056	0.11	14.00 (12.00, 18.00)	14.00 (12.00, 18.00)	15.00 (12.00, 18.00)	0.938	0.029
BUN, mg/dL	23.00 (14.00, 41.25)	33.00 (18.00, 57.00)	22.00 (14.00, 37.00)	<0.001	0.546	29.00 (17.00, 52.00)	31.00 (17.00, 52.00)	28.00 (16.00, 52.00)	0.208	0.029
Potassium, mEq/L	4.20 (3.70, 4.70)	4.40 (3.90, 5.00)	4.10 (3.70, 4.60)	<0.001	0.359	4.30 (3.90, 4.90)	4.40 (3.90, 5.00)	4.30 (3.80, 4.90)	0.087	0.048
Bicarbonate, mmol/L	21.00 (18.00, 24.00)	22.00 (18.00, 24.00)	21.00 (18.00, 24.00)	0.988	0.027	22.00 (18.00, 25.00)	22.00 (18.00, 24.75)	21.00 (18.00, 24.75)	0.972	0.017
Calcium, mg/dL	8.20 (7.70, 8.70)	8.40 (7.90, 8.90)	8.20 (7.60, 8.70)	<0.001	0.223	8.30 (7.80, 8.90)	8.40 (7.90, 8.90)	8.30 (7.73, 8.90)	0.156	0.01
Chloride, mEq/L	103.00 (98.00, 107.00)	103.00 (97.00, 107.00)	103.00 (98.00, 108.00)	0.028	0.107	103.00 (97.00, 107.00)	103.00 (98.00, 107.00)	102.00 (97.00, 107.00)	0.227	0.074
ALT, U/L	33.00 (18.00, 72.00)	36.00 (19.00, 72.00)	32.00 (18.00, 72.00)	0.182	0.004	35.00 (18.00, 76.25)	35.00 (18.25, 71.75)	35.00 (17.00, 80.00)	0.958	0.017
ALP, U/L	100.00 (75.00, 152.00)	106.00 (75.00, 162.50)	99.00 (74.00, 148.00)	0.034	0.127	103.00 (75.00, 154.00)	105.50 (75.00, 157.75)	100.50 (75.00, 145.00)	0.257	0.091
AST, U/L	67.00 (37.00, 158.00)	65.00 (37.00, 146.00)	68.00 (37.00, 159.00)	0.605	0.001	66.00 (34.75, 160.00)	64.00 (36.00, 141.75)	70.00 (33.25, 170.50)	0.406	0.011
Total Bilirubin, mg/dL	2.40 (1.10, 6.00)	2.70 (1.20, 7.85)	2.40 (1.10, 5.80)	0.004	0.285	2.60 (1.10, 7.00)	2.60 (1.10, 7.47)	2.50 (1.10, 6.60)	0.4	0.088
Magnesium, mg/dL	1.90 (1.70, 2.20)	2.10 (1.90, 2.40)	1.80 (1.60, 2.10)	<0.001	0.752	2.00 (1.90, 2.30)	2.10 (1.90, 2.30)	2.00 (1.80, 2.40)	0.004	0.043
HR, Beats/min	86.67 (75.77, 98.53)	84.19 (71.64, 95.41)	87.36 (77.07, 99.39)	<0.001	0.223	84.28 (72.66, 96.04)	84.20 (71.74, 95.83)	84.48 (74.15, 96.36)	0.452	0.036
MBP, mmHg	75.05 (69.07, 83.11)	73.37 (67.22, 82.02)	75.65 (69.61, 83.43)	<0.001	0.215	73.92 (67.58, 82.55)	73.74 (67.64, 82.57)	74.33 (67.51, 82.45)	0.629	0.007
RR,Times/min	18.23 (15.92, 21.24)	18.00 (15.71, 21.07)	18.30 (16.02, 21.28)	0.085	0.07	18.04 (15.75, 20.86)	18.00 (15.73, 21.05)	18.09 (15.78, 20.73)	0.915	0.01
Temperature, °C	36.79 (36.57, 37.04)	36.69 (36.44, 36.90)	36.82 (36.61, 37.06)	<0.001	0.396	36.72 (36.48, 36.93)	36.70 (36.46, 36.91)	36.74 (36.50, 36.96)	0.043	0.073
Spo2, %	96.95 (95.38, 98.42)	96.61 (95.07, 98.14)	97.04 (95.46, 98.48)	<0.001	0.287	96.69 (95.12, 98.21)	96.67 (95.09, 98.18)	96.76 (95.14, 98.26)	0.526	0.01
Weight, Kg	82.55 (70.00, 98.10)	85.60 (72.35, 100.25)	81.80 (69.30, 97.20)	<0.001	0.121	84.85 (71.44, 99.31)	86.05 (72.67, 100.07)	83.50 (70.40, 97.45)	0.014	0.114
APSIII score	51.00 (38.00, 70.00)	54.00 (39.00, 75.00)	50.00 (38.00, 68.00)	0.002	0.15	52.00 (38.00, 72.00)	52.00 (38.00, 73.00)	52.00 (39.00, 71.75)	0.957	0.022
Charlson comorbidity index	5.00 (4.00, 7.00)	6.00 (4.00, 8.00)	5.00 (4.00, 7.00)	<0.001	0.253	6.00 (4.00, 8.00)	6.00 (4.00, 8.00)	6.00 (4.00, 8.00)	0.659	0.017
GCS score	15.00 (15.00, 15.00)	15.00 (14.00, 15.00)	15.00 (15.00, 15.00)	<0.001	0.078	15.00 (15.00, 15.00)	15.00 (15.00, 15.00)	15.00 (15.00, 15.00)	0.142	0.02
MELD score	22.00 (14.00, 30.34)	24.53 (15.82, 33.00)	21.60 (14.00, 29.70)	<0.001	0.215	23.56 (15.00, 31.65)	23.09 (15.00, 32.11)	23.69 (15.00, 31.00)	0.75	0.022
OASIS score	32.00 (26.00, 38.00)	31.00 (25.00, 37.00)	32.00 (26.00, 38.00)	0.005	0.097	31.00 (25.00, 37.00)	31.00 (25.00, 37.00)	30.00 (25.00, 37.00)	0.979	0.006
SAPSII score	37.00 (29.00, 48.25)	40.00 (30.00, 52.00)	37.00 (28.00, 47.00)	<0.001	0.203	39.00 (30.00, 50.00)	39.00 (29.00, 52.00)	39.00 (30.00, 50.00)	0.883	0.024
SOFA score	3.00 (1.00, 6.00)	3.00 (1.00, 7.00)	3.00 (1.00, 6.00)	0.311	0.097	3.00 (1.00, 6.00)	3.00 (1.00, 6.00)	3.00 (1.00, 6.00)	0.915	0.035
Los Hospital, days	8.47 (4.74, 15.97)	5.87 (3.17, 10.29)	9.46 (5.26, 17.66)	<0.001	0.403	7.05 (3.94, 13.13)	5.82 (3.19, 10.08)	8.67 (4.89, 15.84)	<0.001	0.341
Los Icu, days	2.28 (1.20, 4.72)	1.59 (0.93, 3.01)	2.59 (1.35, 5.18)	<0.001	0.345	1.87 (1.02, 3.74)	1.59 (0.93, 3.04)	2.08 (1.17, 4.18)	<0.001	0.244
In-hospital mortality, N (%)				<0.001	0.291				<0.001	0.218
Alive	2,533 (76.48)	472 (67.14)	2061 (79.00)			961 (73.47)	451 (68.96)	510 (77.98)		
Dead	779 (23.52)	231 (32.86)	548 (21.00)			347 (26.53)	203 (31.04)	144 (22.02)		
Gender, N (%)				0.017	0.101				0.905	0.007
Female	1,139 (34.39)	215 (30.58)	924 (35.42)			416 (31.8)	207 (31.65)	209 (31.96)		
Male	2,173 (65.61)	488 (69.42)	1,685 (64.58)			892 (68.2)	447 (68.35)	445 (68.04)		
Race, N (%)				0.182	0.095				0.474	0.088
Black	230 (6.94)	41 (5.83)	189 (7.24)			89 (6.8)	41 (6.27)	48 (7.34)		
White	2,198 (66.36)	475 (67.57)	1723 (66.04)			893 (68.27)	442 (67.58)	451 (68.96)		
Other	446 (13.47)	105 (14.94)	341 (13.07)			176 (13.46)	97 (14.83)	79 (12.08)		
Unknown	438 (13.22)	82 (11.66)	356 (13.65)			150 (11.47)	74 (11.31)	76 (11.62)		
180-day mortality, N (%)				<0.001	0.299				0.005	0.155
Alive	1992 (60.14)	343 (48.79)	1,649 (63.20)			716 (54.74)	333 (50.92)	383 (58.56)		
Dead	1,320 (39.86)	360 (51.21)	960 (36.80)			592 (45.26)	321 (49.08)	271 (41.44)		
Myocardial Infarction, N (%)				0.057	0.079				0.382	0.046
No	3,058 (92.33)	661 (94.03)	2,397 (91.87)			1,218 (93.12)	613 (93.73)	605 (92.51)		
Yes	254 (7.67)	42 (5.97)	212 (8.13)			90 (6.88)	41 (6.27)	49 (7.49)		
Congestive heart failure, N (%)				0.087	0.072				0.655	0.024
No	2,721 (82.16)	593 (84.35)	2,128 (81.56)			1,092 (83.49)	549 (83.94)	543 (83.03)		
Yes	591 (17.84)	110 (15.65)	481 (18.44)			216 (16.51)	105 (16.06)	111 (16.97)		
Peripheral vascular disease, N (%)				0.163	0.058				1	0
No	3,105 (93.75)	667 (94.88)	2,438 (93.45)			1,236 (94.5)	618 (94.50)	618 (94.50)		
Yes	207 (6.25)	36 (5.12)	171 (6.55)			72 (5.5)	36 (5.50)	36 (5.50)		
Chronic pulmonary disease, N (%)				0.766	0.013				0.384	0.049
No	2,586 (78.08)	546 (77.67)	2040 (78.19)			1,023 (78.21)	505 (77.22)	518 (79.20)		
Yes	726 (21.92)	157 (22.33)	569 (21.81)			285 (21.79)	149 (22.78)	136 (20.80)		
Diabetes, N (%)				0.022	0.098				0.953	0.003
No	2,330 (70.35)	470 (66.86)	1860 (71.29)			881 (67.35)	441 (67.43)	440 (67.28)		
Yes	982 (29.65)	233 (33.14)	749 (28.71)			427 (32.65)	213 (32.57)	214 (32.72)		
Renal disease, N (%)				<0.001	0.227				0.455	0.042
No	2,788 (84.18)	548 (77.95)	2,240 (85.86)			1,033 (78.98)	511 (78.13)	522 (79.82)		
Yes	524 (15.82)	155 (22.05)	369 (14.14)			275 (21.02)	143 (21.87)	132 (20.18)		
Malignant cancer, N (%)				<0.001	0.205				0.642	0.026
No	2,776 (83.82)	549 (78.09)	2,227 (85.36)			1,017 (77.75)	512 (78.29)	505 (77.22)		
Yes	536 (16.18)	154 (21.91)	382 (14.64)			291 (22.25)	142 (21.71)	149 (22.78)		
Hypertension, N (%)				<0.001	0.196				0.495	0.038
No	2,785 (84.09)	553 (78.66)	2,232 (85.55)			1,038 (79.36)	514 (78.59)	524 (80.12)		
Yes	527 (15.91)	150 (21.34)	377 (14.45)			270 (20.64)	140 (21.41)	130 (19.88)		
Sepsis, N (%)				<0.001	0.203				0.591	0.029
No	2,371 (71.59)	555 (78.95)	1816 (69.61)			1,026 (78.44)	517 (79.05)	509 (77.83)		
Yes	941 (28.41)	148 (21.05)	793 (30.39)			282 (21.56)	137 (20.95)	145 (22.17)		
Ascites, N (%)				0.296	0.044				0.824	0.012
No	1829 (55.22)	376 (53.49)	1,453 (55.69)			726 (55.5)	361 (55.20)	365 (55.81)		
Yes	1,483 (44.78)	327 (46.51)	1,156 (44.31)			582 (44.5)	293 (44.80)	289 (44.19)		
HPS, N (%)				0.787	0.011				0.403	0.053
No	3,271 (98.76)	695 (98.86)	2,576 (98.74)			1,295 (99.01)	646 (98.78)	649 (99.24)		
Yes	41 (1.24)	8 (1.14)	33 (1.26)			13 (0.99)	8 (1.22)	5 (0.76)		
CRRT, N (%)				0.004	0.119				0.387	0.046
No	3,002 (90.64)	657 (93.46)	2,345 (89.88)			1,216 (92.97)	612 (93.58)	604 (92.35)		
Yes	310 (9.36)	46 (6.54)	264 (10.12)			92 (7.03)	42 (6.42)	50 (7.65)		
Invasive ventilation, N (%)				<0.001	0.175				0.666	0.024
No	777 (23.46)	205 (29.16)	572 (21.92)			363 (27.75)	185 (28.29)	178 (27.22)		
Yes	2,535 (76.54)	498 (70.84)	2037 (78.08)			945 (72.25)	469 (71.71)	476 (72.78)		
HS, N (%)				<0.001	0.253				0.757	0.017
No	2,941 (88.8)	583 (82.93)	2,358 (90.38)			1,112 (85.02)	554 (84.71)	558 (85.32)		
Yes	371 (11.2)	120 (17.07)	251 (9.62)			196 (14.98)	100 (15.29)	96 (14.68)		
HE, N (%)				<0.001	0.251				0.743	0.018
No	3,014 (91)	603 (85.78)	2,411 (92.41)			1,136 (86.85)	570 (87.16)	566 (86.54)		
Yes	298 (9)	100 (14.22)	198 (7.59)			172 (13.15)	84 (12.84)	88 (13.46)		
PH, N (%)				0.636	0.02				0.867	0.009
No	1849 (55.83)	398 (56.61)	1,451 (55.62)			731 (55.89)	367 (56.12)	364 (55.66)		
Yes	1,463 (44.17)	305 (43.39)	1,158 (44.38)			577 (44.11)	287 (43.88)	290 (44.34)		
Diarrhea, N (%)				0.004	0.116				0.001	0.154
No	3,143 (94.9)	682 (97.01)	2,461 (94.33)			1,247 (95.34)	636 (97.25)	611 (93.43)		
Yes	169 (5.1)	21 (2.99)	148 (5.67)			61 (4.66)	18 (2.75)	43 (6.57)		
SBP, N (%)				0.083	0.075				0.851	0.01
No	3,031 (91.52)	632 (89.90)	2,399 (91.95)			1,182 (90.37)	590 (90.21)	592 (90.52)		
Yes	281 (8.48)	71 (10.10)	210 (8.05)			126 (9.63)	64 (9.79)	62 (9.48)		
AKI, N (%)				0.028	0.094				0.225	0.066
No	823 (24.85)	197 (28.02)	626 (23.99)			386 (29.51)	183 (27.98)	203 (31.04)		
Yes	2,489 (75.15)	506 (71.98)	1983 (76.01)			922 (70.49)	471 (72.02)	451 (68.96)		

### 3.2 Magnesium sulfate and primary outcomes

In the matched cohort, the in-hospital all-cause mortality rate was 22.02% (144 of 654 patients) for patients who received magnesium sulfate, compared with 31.04% (203 of 654 patients) for those who did not receive magnesium sulfate. This difference between the two groups was statistically significant (p < 0.001) as detailed in Table 1. Further univariate logistic regression analysis (OR = 0.63, 95% CI 0.49-0.80, p < 0.001) and multivariate logistic regression analysis (OR = 0.47, 95% CI 0.33-0.69, p < 0.001) showed that magnesium sulfate use was significantly associated with a reduced risk of in-hospital all-cause mortality. Both univariate Cox analysis (HR = 0.76, 95% CI 0.65-0.89, p < 0.001) and multivariate Cox analysis (HR = 0.61, 95% CI 0.51-0.72, p < 0.001) showed that magnesium sulfate use was associated with a reduced risk of all-cause mortality within 180 days. See Table 2 for details.

**TABLE 2:** Association between magnesium sulfate and clinical outcomes in cirrhosis.

	Model 1		Model 2	
	OR/HR (95%CI)	*P*	OR/HR (95%CI)	*P*
In-hospital all-cause mortality				
Magnesium sulfate				
No	1.00 (Reference)		1.00 (Reference)	
Yes	0.63 (0.49–0.80)	<0.001	0.47 (0.33–0.69)	<0.001
180-day all-cause mortality				
Magnesium sulfate				
No	1.00 (Reference)		1.00 (Reference)	
Yes	0.76 (0.65–0.89)	<0.001	0.61 (0.51–0.72)	<0.001

OR, Odds Ratio; HR, Hazard Ratio; CI, Confidence Interval. Model 1: Crude. Model 2: Adjust: age, gender, rbc, wbc, platelet, sodium, creatinine, bun, potassium, bicarbonate, calcium, chloride, magnesium, HR, MBP, RR, temperature, Spo2, myocardial_infarction, congestive_heart_failure, chronic_pulmonary_disease, diabetes, peripheral_vascular_disease, renal_disease, malignant_cancer, diarrhea, SAPSII score, MELD score, SOFA score, sepsis, Charlson_Comorbidity_Index, hypertension, ascites, HPS, CRRT, invasive ventilation, HS, HE, PH, SBP, AKI.

### 3.3 Magnesium sulfate and secondary outcomes

**FIGURE 2:**
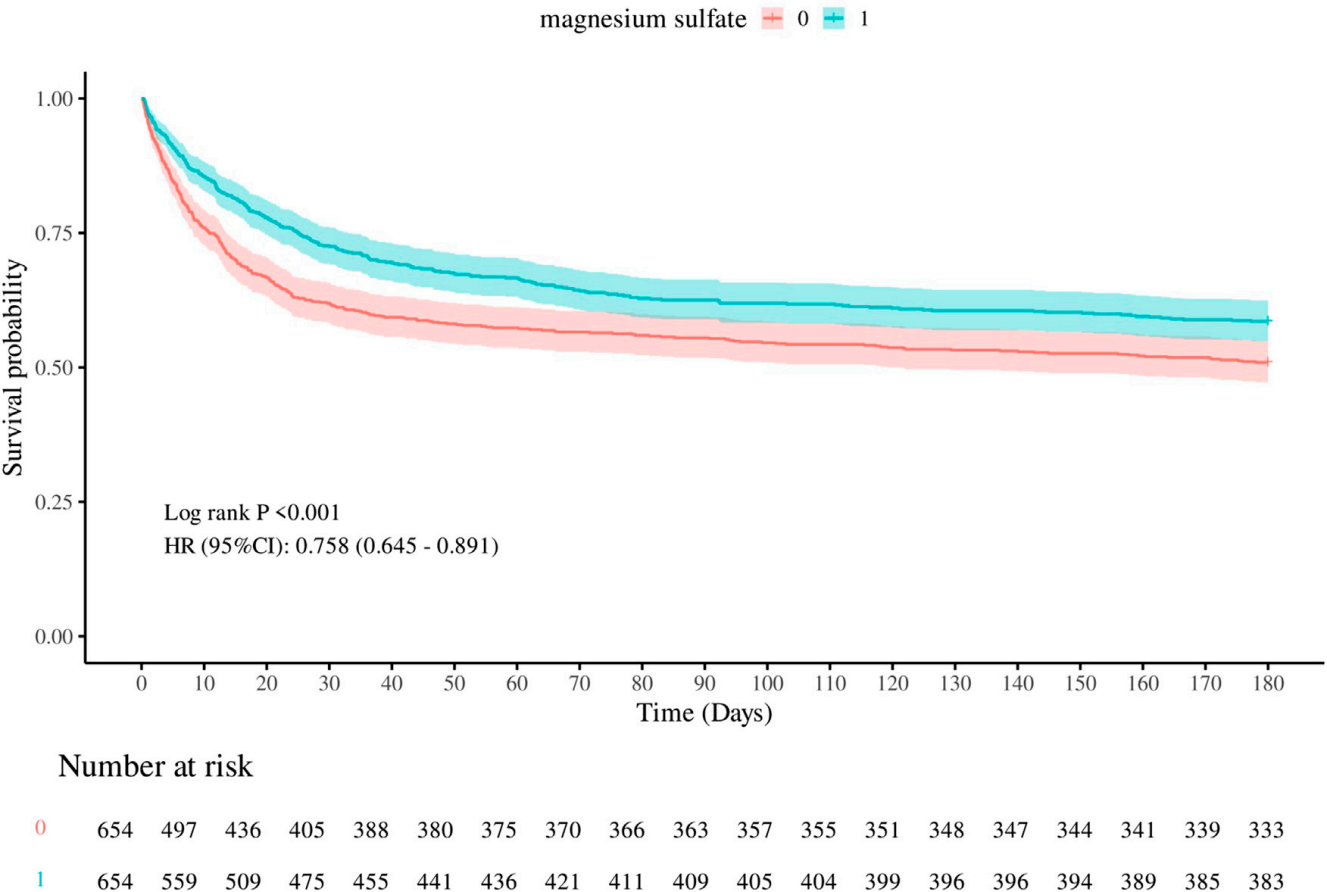
Kaplan-Meier survival curves of 180-day all-cause mortality. 0: Magnesium sulfate not used 1: Magnesium sulfate used.

In the matched cohort, the 180-day all-cause mortality rate was 41.44% (271/654) in the cohort using magnesium sulfate and 49.08% (321/654) in the cohort not using magnesium sulfate (p = 0.005). Figure 2 shows the Kaplan-Meier curves of 180-day all-cause mortality stratified by magnesium sulfate use in the matched cohort. Cox regression analysis showed that in the matched cohort, both univariate analysis (HR, 0.76; 95% CI, 0.65–0.89; p < 0.001) and multivariate analysis (HR, 0.61; 95% CI, 0.51–0.72; p < 0.001) showed that the use of magnesium sulfate was associated with a reduced 180-day all-cause mortality rate.

### 3.4 Subgroup analysis

**FIGURE 3:**
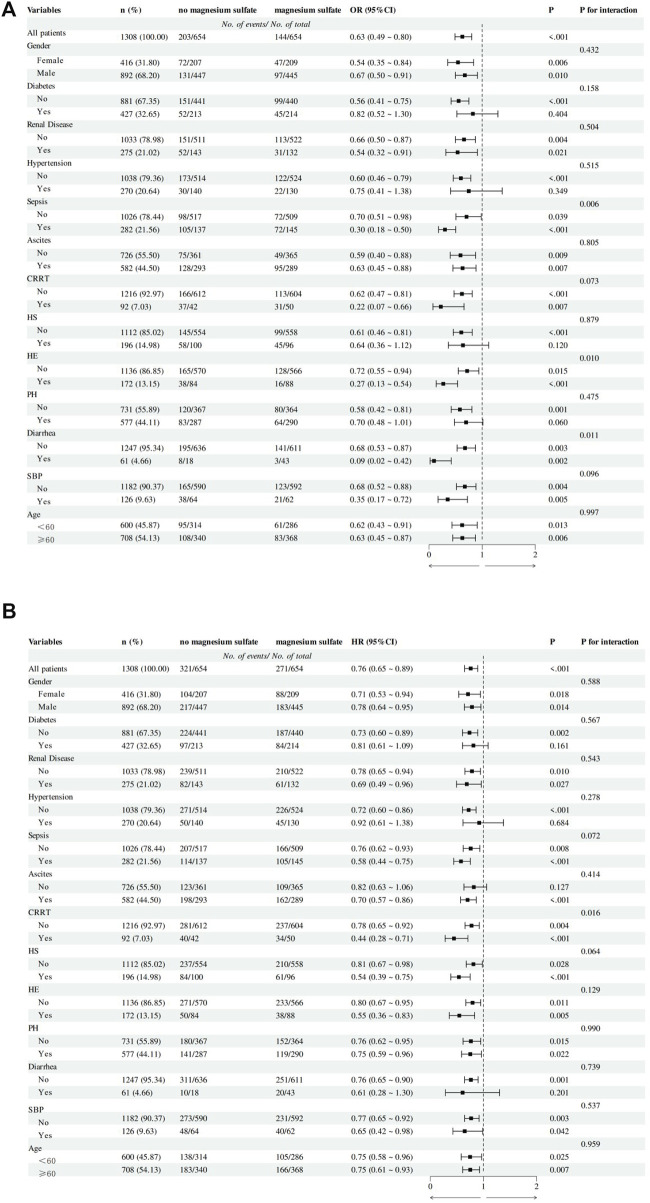
Subgroup analysis of the association between magnesium sulfate use and outcomes in critically ill patients with cirrhosis. **(A)** In-hospital mortality logistics regression subgroup analysis forest plot. **(B)** 180-day mortality Cox regression subgroup analysis forest plot. Abbreviation: CRRT, Continuous Renal Replacement Therapy; HS, Hepatorenal syndrome; HE, Hepatic encephalopathy; PH, Portal hypertension; SBP, Spontaneous bacterial peritonitis.

Figure 3 shows the results of subgroup analysis of in-hospital all-cause mortality and 180-day all-cause mortality in the matched cohort. In the subgroup analysis of in-hospital all-cause mortality, we found significant interactions between magnesium sulfate use and sepsis, hepatic encephalopathy, and diarrhea subgroups (P for interaction less than 0.05), but no significant interactions with other stratification variables (P for interaction >0.05).

In the subgroup analysis of 180-day all-cause mortality, we found that magnesium sulfate use continued to show significant interactions in the CRRT subgroup (P for interaction = 0.016), and there was no interaction between other stratification variables and magnesium sulfate exposure (P for interaction >0.05).

### 3.5 Sensitivity analyses

#### 3.5.1 Whole cohort

In the unmatched cohort, logistic regression analysis showed that in-hospital all-cause mortality was significantly inversely correlated with the use of magnesium sulfate. Both univariate analysis (OR, 0.54; 95% CI, 0.45–0.65; p < 0.001) and multivariate analysis (OR, 0.48; 95% CI, 0.37–0.64; p < 0.001) supported this conclusion. More detailed Cox regression analysis results, 180-day all-cause mortality Kaplan-Meier curves stratified by magnesium sulfate use, and subgroup analysis results are shown in Supplementary Material S5–S7.

#### 3.5.2 Excluding sepsis cohort before PSM

Excluding the sepsis cohort, logistic regression analysis revealed a significant negative correlation between magnesium sulfate usage and in-hospital all-cause mortality. This conclusion was supported by both univariate analysis (OR, 0.49; 95% CI, 0.39–0.64; p < 0.001) and multivariate analysis (OR, 0.67; 95% CI, 0.46–0.97; p = 0.036). Further details regarding Cox regression analysis, Kaplan-Meier curves for 180-day all-cause mortality stratified by magnesium sulfate usage, and subgroup analysis results can be found in Supplementary Material S8–S10.

#### 3.5.3 Cohort excluding patients who received oral magnesium preparations

When the cohort was restricted to exclude oral magnesium ion preparations, logistic regression analysis demonstrated a significant inverse relationship between magnesium sulfate administration and in-hospital all-cause mortality. This finding was consistent across both univariate analysis (OR, 0.53; 95% CI, 0.44–0.64; p < 0.001) and multivariate analysis (OR, 0.48; 95% CI, 0.36–0.64; p < 0.001). Supplementary Material S11–S13 provide additional details, including Cox regression analysis results, Kaplan-Meier curves for 180-day all-cause mortality based on magnesium sulfate usage, and subgroup analysis outcomes.

## 4 Discussion

We conducted a retrospective cohort study based on the MIMIC-IV3.0 database and showed that magnesium sulfate use was associated with reduced in-hospital and 180-day all-cause mortality in critically ill patients with cirrhosis admitted to the ICU. This association was consistent in the propensity score-matched cohort and in sensitivity analyses, demonstrating the robustness of this finding. To our knowledge, this is the first retrospective study to explore the association between magnesium supplementation and outcomes in critically ill cirrhotic patients in the ICU using real-world data.

### 4.1 Possible explanations for findings

The following reasons may explain why magnesium sulfate use is associated with improved in-hospital and 180-day mortality in patients with critically ill cirrhosis. Magnesium plays a vital role in various physiological processes such as anti-inflammatory response and regulation of oxidative stress (De Baaij et al., 2015; Veronese et al., 2022). In terms of anti-inflammatory effects, magnesium deficiency leads to activation of phagocytes and weakens the calcium channel blocking effect, resulting in increased intracellular calcium concentration and activation of NMDA receptors, which in turn activates cellular inflammatory responses and releases a large amount of inflammatory mediators (Romani, 2013). In addition, magnesium deficiency can activate NF-κB, leading to an aggravated inflammatory response (Kabe et al., 2005). Studies have shown that magnesium supplementation is associated with improvements in inflammatory factors (Eidi et al., 2013). A meta-analysis showed that magnesium supplementation could significantly reduce the levels of inflammatory markers such as CRP in serum, suggesting that magnesium supplementation has the potential to alleviate inflammatory responses (Veronese et al., 2022). In terms of oxidative stress regulation, magnesium deficiency may lead to increased activity of mitochondrial respiratory chain enzymes, resulting in increased reactive oxygen species (ROS) levels and increased oxidative stress (Liu et al., 2007). Magnesium supplementation can inhibit excessive mitochondrial ROS production (Liu et al., 2020). A study from South Korea showed that magnesium salvia miltiorrhiza B (MLB) can inhibit the production of NF-kB and reduce reactive oxygen species (ROS) produced by hepatic stellate cells (Paik et al., 2011). Other studies have shown that magnesium supplementation can improve mitochondrial function and reduce oxidative stress (Liu et al., 2019). Under low magnesium conditions, DNA is more susceptible to ROS damage, while under high magnesium conditions, DNA is less likely to be damaged by ROS. This mechanism may be related to the ability of magnesium to covalently bind to DNA, thereby stabilizing the double helix structure (De Baaij et al., 2015).

In the subgroup analysis of the primary outcome, we found that the use of magnesium sulfate was more effective in reducing the in-hospital mortality of patients with cirrhosis with sepsis, hepatic encephalopathy, and diarrhea. The possible explanations are as follows: 1. Compared with cirrhotic patients without sepsis, cirrhotic patients with sepsis have more severe inflammation in their bodies, thus making full use of the anti-inflammatory effect of magnesium ions. This is consistent with the results of a previous study (Gu et al., 2023). 2. Compared with cirrhotic patients without hepatic encephalopathy, cirrhotic patients with hepatic encephalopathy have worse liver function. Some studies have shown that the degree of magnesium ion deficiency may be related to the severity of cirrhosis in patients with cirrhosis (Nangliya et al., 2015; Chaudhry et al., 2018; Llibre-Nieto et al., 2021). Therefore, the benefits of magnesium sulfate supplementation may be more prominent. 3. Considering that diarrhea may aggravate electrolyte imbalance (include magnesium ion loss), the degree of magnesium deficiency is more severe in patients with cirrhosis and diarrhea, so the protective effect of magnesium sulfate supplementation is significantly enhanced.

### 4.2 Implications for clinical practice

In this study, we found that the use of magnesium sulfate may be beneficial for the prognosis of patients with cirrhosis. The results of the subgroup analysis of the primary outcome suggest that for patients with cirrhosis who have hepatic encephalopathy, sepsis, and diarrhea, blood magnesium should be actively monitored and magnesium supplementation should be given priority (potentially with greater survival benefits). For patients who do not have hepatic encephalopathy, sepsis, or diarrhea, magnesium supplementation still has a protective effect, but the degree of benefit needs to be weighed against the risks. However, due to the limited sample size of the subgroup, this result may be due to chance and should be interpreted with caution. When magnesium sulfate is overdosed intravenously, nausea, dizziness, weakness and confusion, drowsiness and weakened reflexes may occur, as well as headaches, flushing, urinary complications and gastrointestinal symptoms caused by bladder paralysis (Jahnen-Dechent and Ketteler, 2012; Aal-Hamad et al., 2023). Blurred vision and mild decrease in blood pressure may also occur (Chang et al., 2014; De Baaij et al., 2015). The safety of magnesium sulfate is highly dependent on dose control, administration rate and dynamic monitoring. Clinically, it is necessary to strictly grasp the indications, dynamically evaluate renal function, blood drug concentration and clinical reactions, and avoid empirical medication.

### 4.3 Study limitations

First, since this is a retrospective cohort study, although propensity score matching and multivariate analysis were used to calibrate confounding variables, the results were affected by residual bias and unmeasured confounders, and causal relationships could not be determined. Second, due to the partial missing of medication dose and duration, we failed to evaluate the effect of magnesium sulfate dose on mortality and could only analyze magnesium sulfate medication as a dichotomous variable. Third, due to the diverse indications of magnesium preparations and the fluctuation of blood magnesium concentration during ICU hospitalization, it was difficult for us to calculate the dose of magnesium preparations required for the study group. Fourth, we performed multiple imputations on covariates with a missing rate of less than 25%, which may introduce systematic bias. Finally, our study did not evaluate the safety of magnesium sulfate.

## 5 Conclusion

In patients with cirrhosis admitted to the ICU, magnesium sulfate administration was associated with reduced in-hospital all-cause mortality and 180-day all-cause mortality. Prospective studies are needed to confirm this finding.

## Data Availability

Publicly available datasets were analyzed in this study. These data can be found here: https://physionet.org/content/mimiciv/3.0/.
