# Supplementary material for: The use of magnesium sulfate can reduce the mortality risk of cirrhosis patients: a retrospective cohort study
Source: Front Pharmacol. 2025 Oct 20;16:1551495. doi: 10.3389/fphar.2025.1551495 (PMC12580101; doi:10.3389/fphar.2025.1551495)
Supplement: Supplementary file 2 [file Supplementaryfile2.docx]

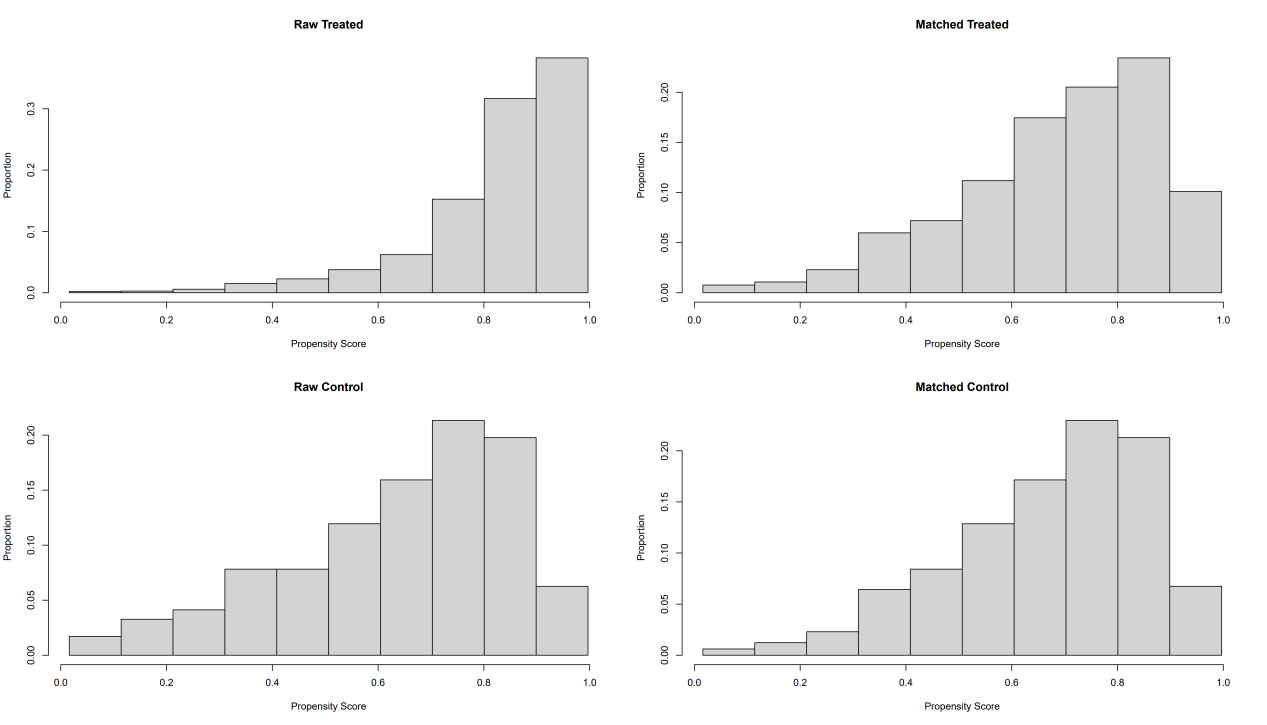


**Supplementary material S2** Distributional balance before and after propensity score matching


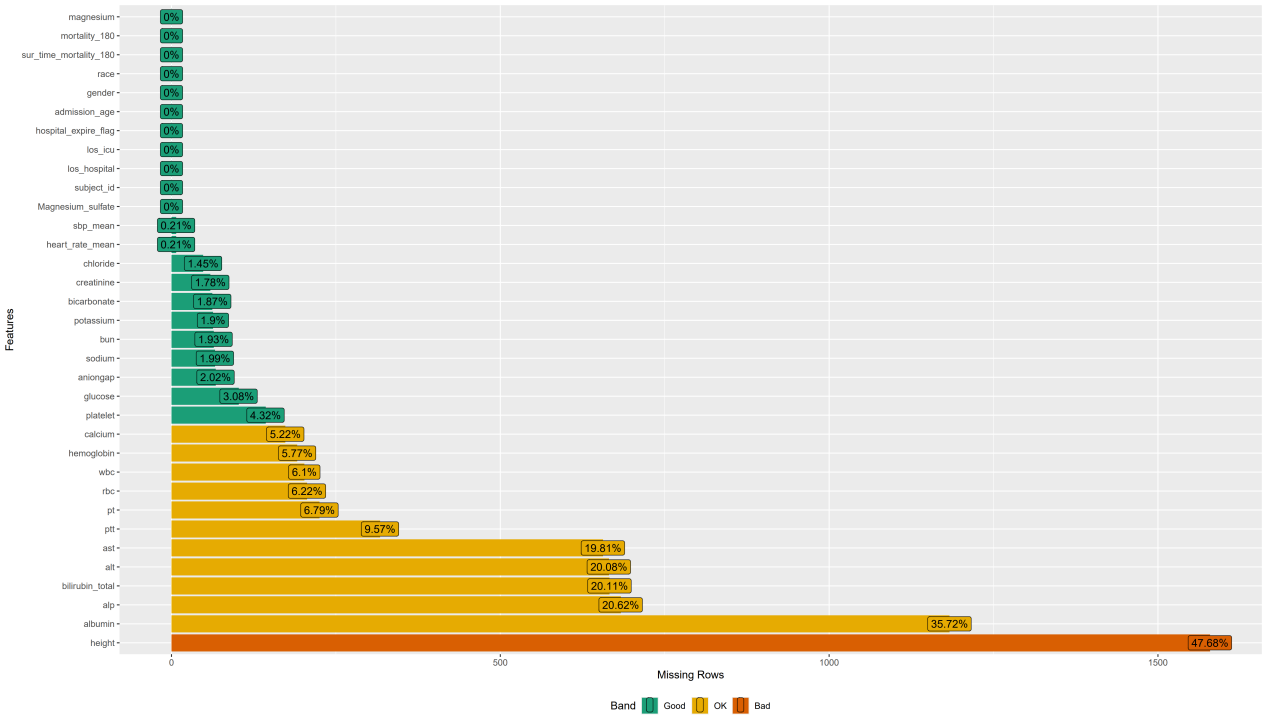


**Supplementary material S3** Percentage of missing data of each variable1


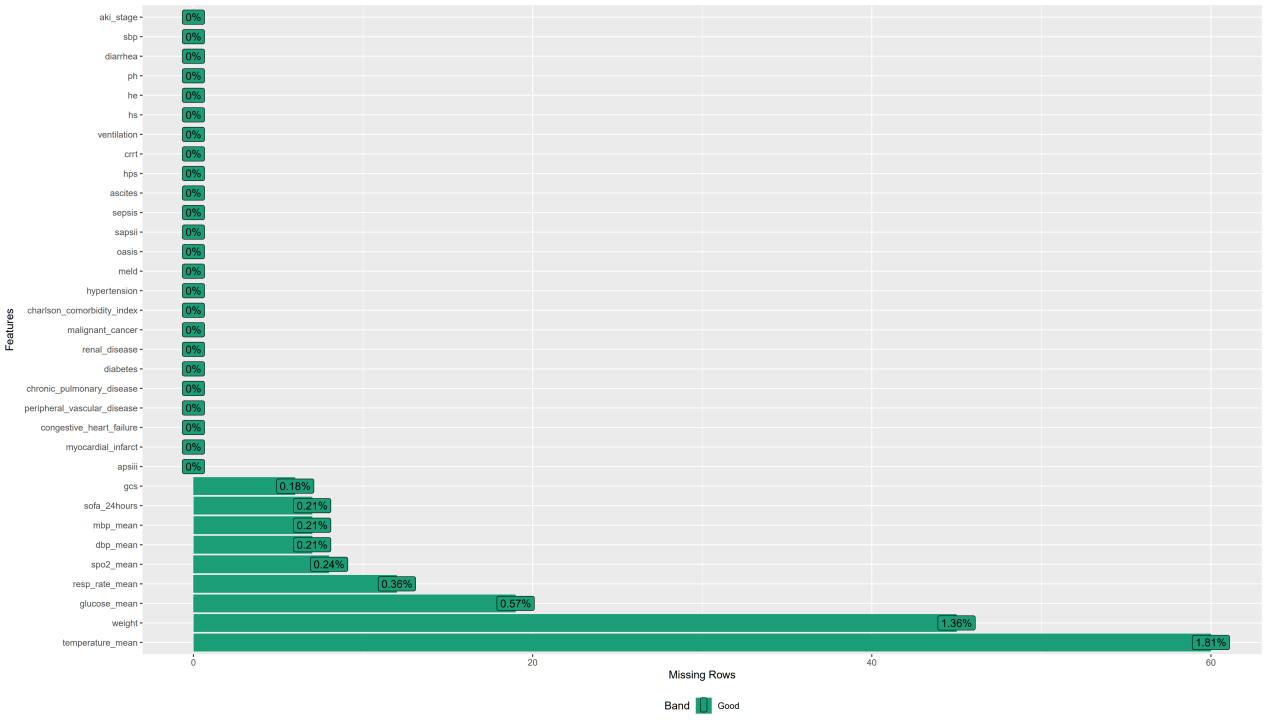


**Supplementary material S3** Percentage of missing data of each variable2


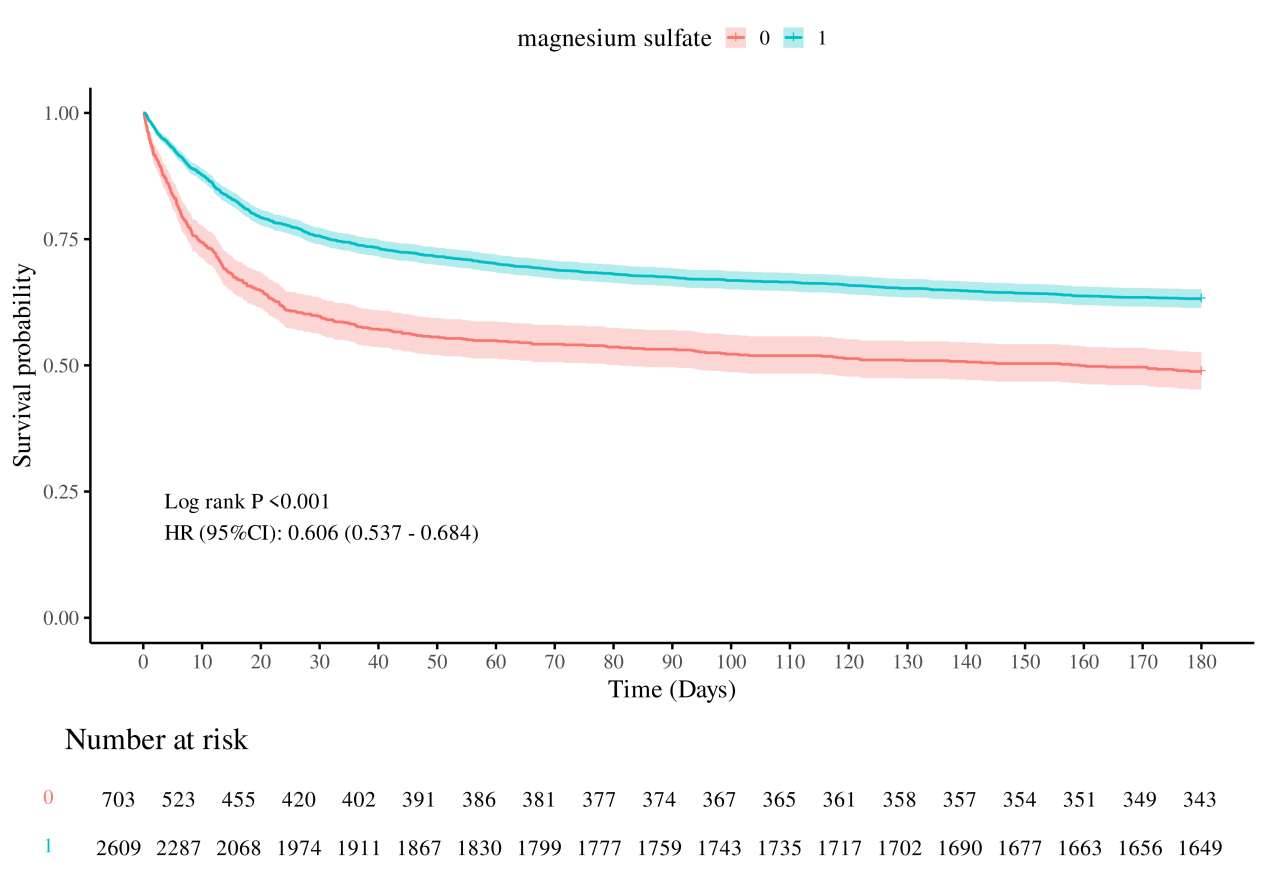


**Supplementary material S6** Kaplan-Meier survival curves of 180-day all-cause mortality. 0:Magnesium sulfate not used 1:Magnesium sulfate used.

(A)


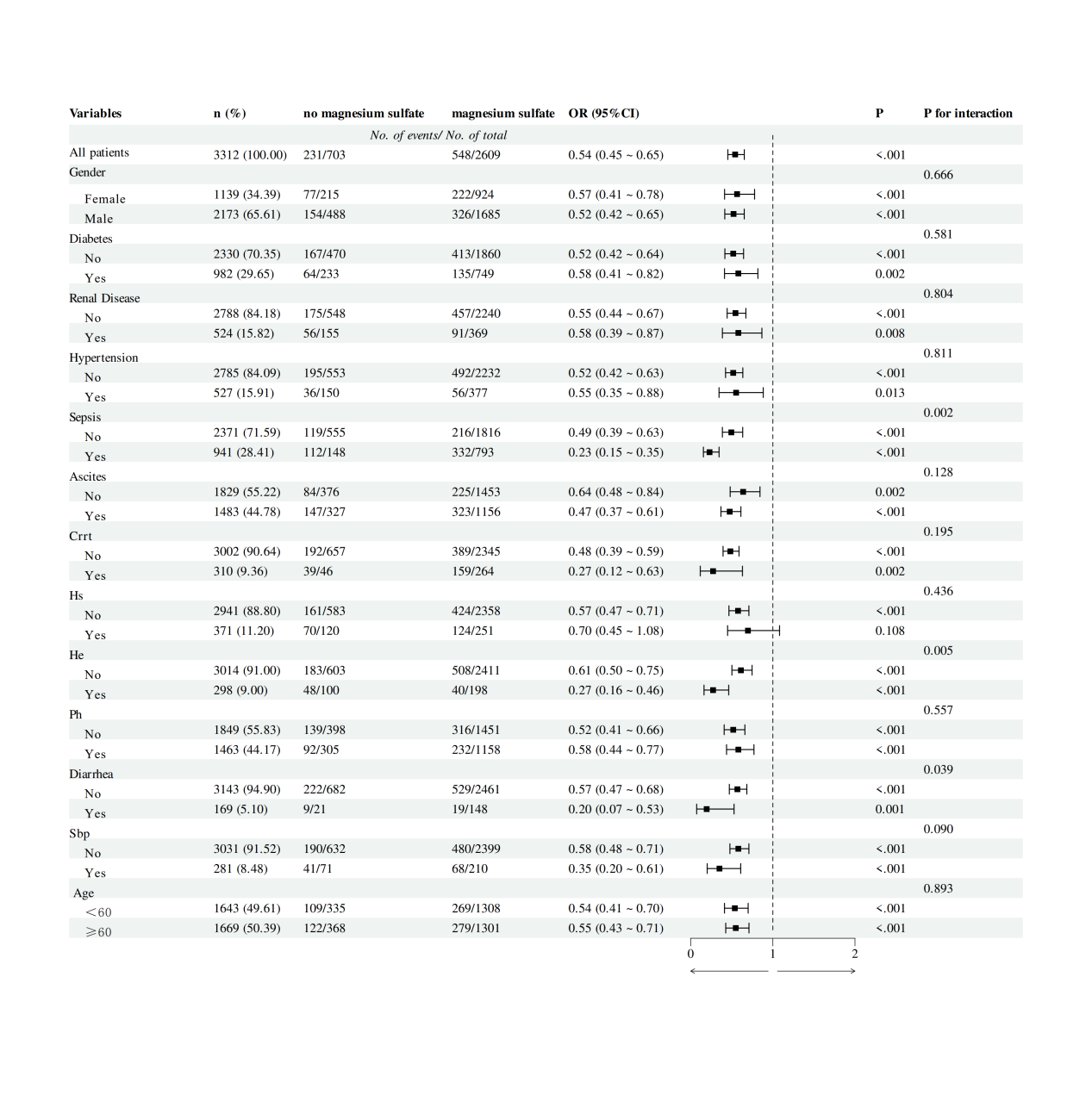


(B)


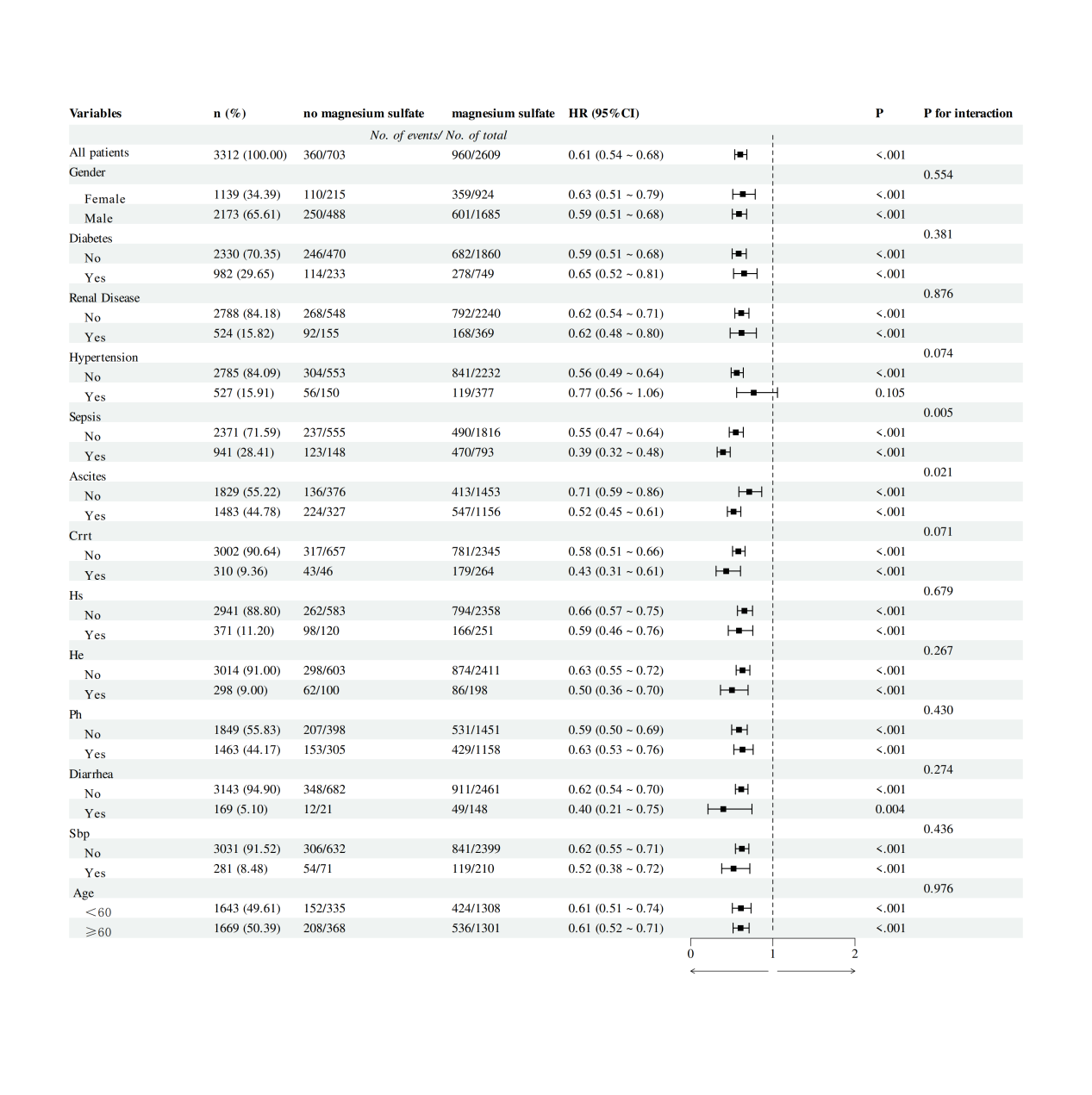


**Supplementary material S7** Subgroup analysis of the association between magnesium sulfate use and outcomes in critically ill patients with cirrhosis.

(A)In-hospital mortality logistics regression subgroup analysis forest plot.

(B)180-day mortality Cox regression subgroup analysis forest plot.

Abbreviation:CRRT, Continuous Renal Replacement Therapy; HS, Hepatorenal syndrome; HE,Hepatic encephalopathy; PH,Portal hypertension; SBP,Spontaneous bacterial peritonitis.


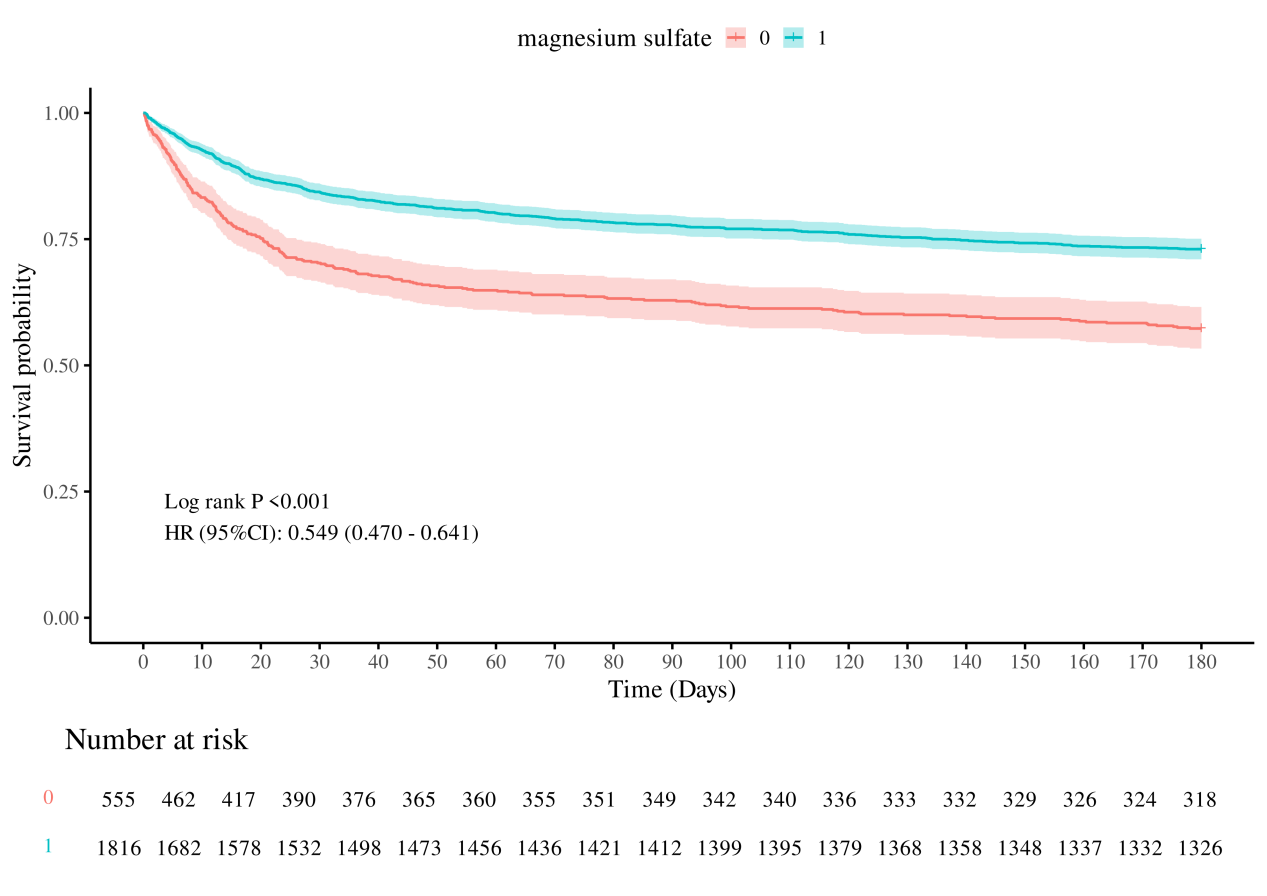


**Supplementary material S9** Kaplan-Meier survival curves of 180-day all-cause mortality. 0:Magnesium sulfate not used 1:Magnesium sulfate used.

(A)


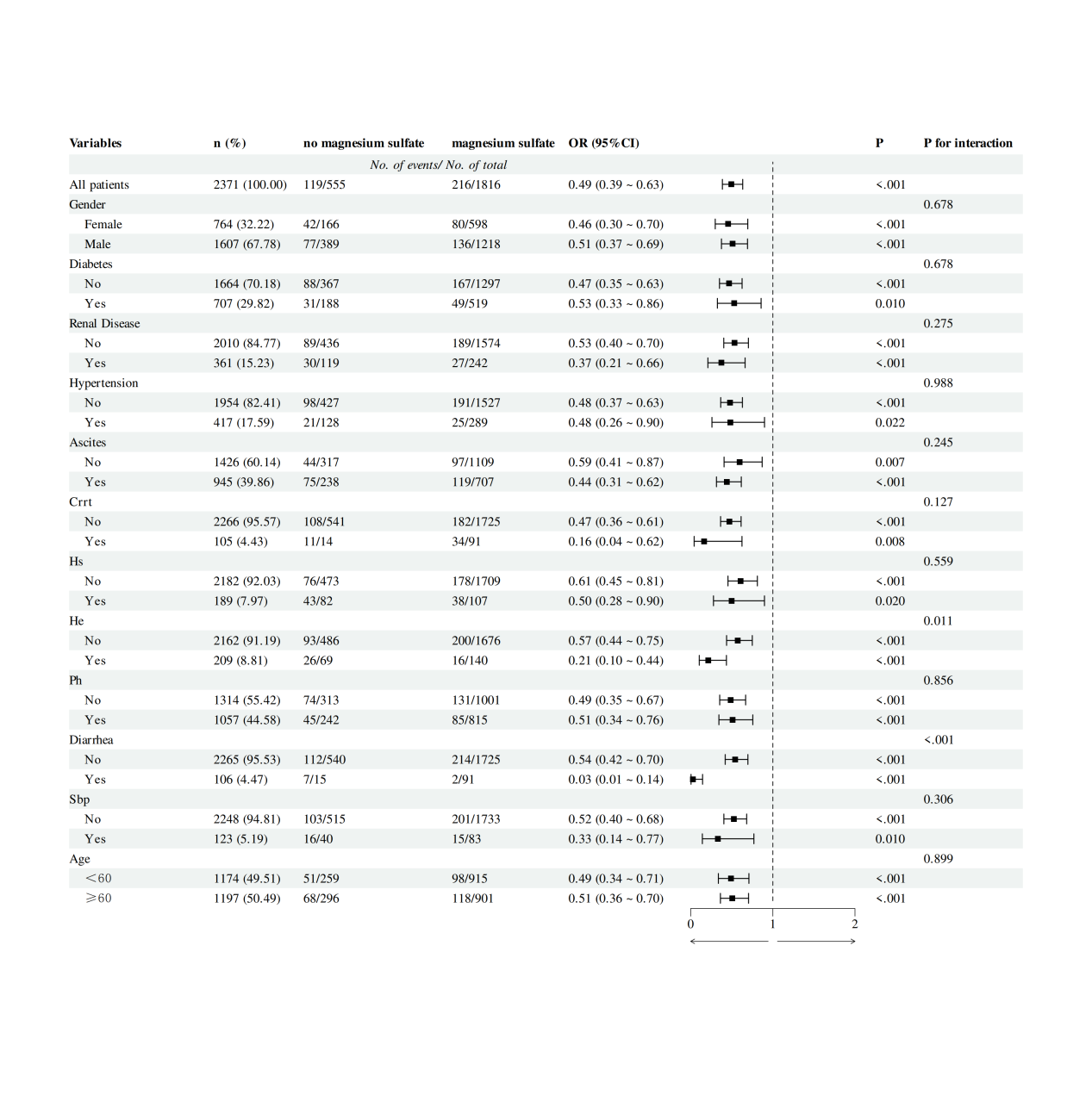


(B)


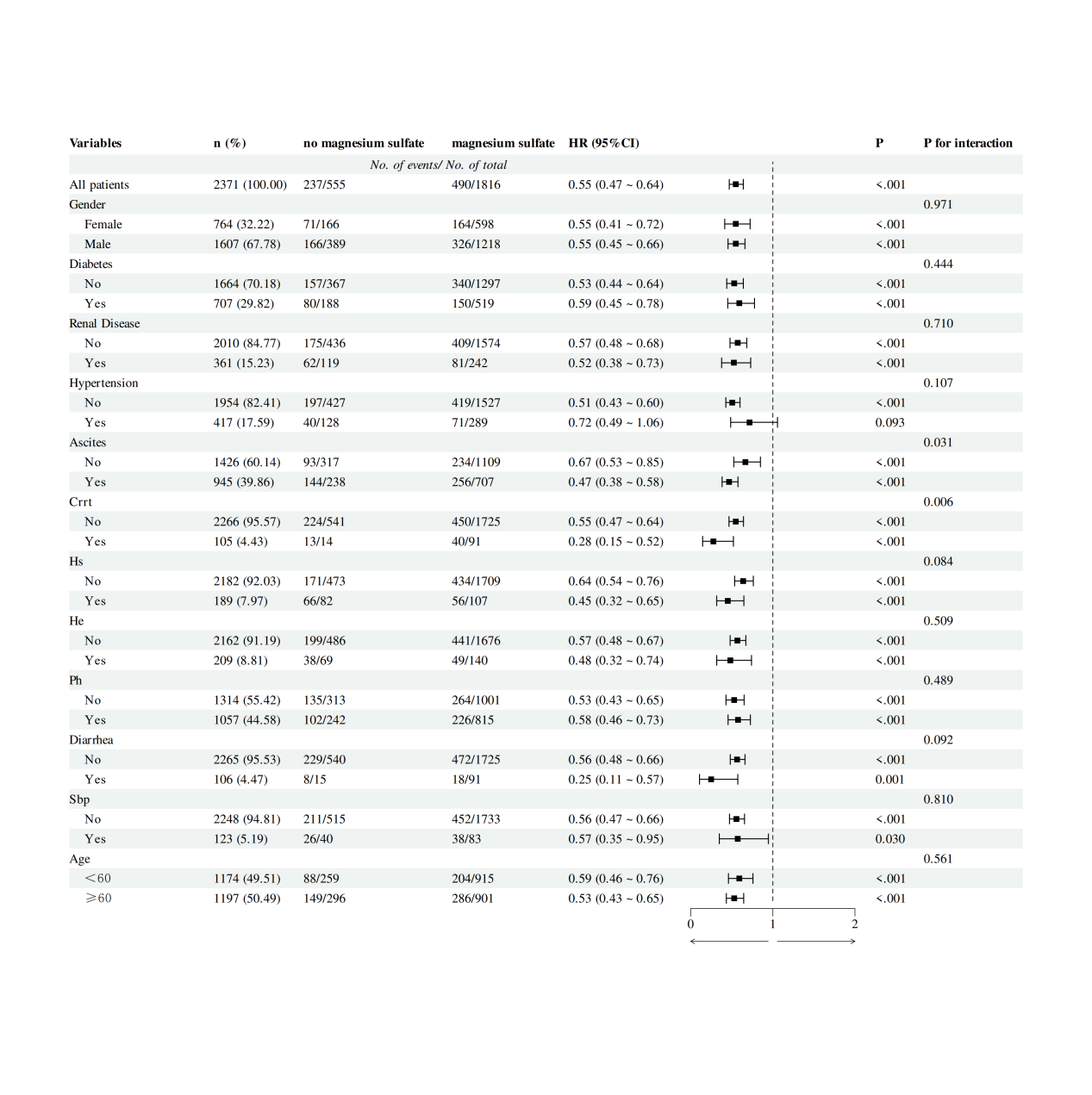


**Supplementary material S10** Subgroup analysis of the association between magnesium sulfate use and outcomes in critically ill patients with cirrhosis.

(A)In-hospital mortality logistics regression subgroup analysis forest plot.

(B)180-day mortality Cox regression subgroup analysis forest plot.

Abbreviation:CRRT, Continuous Renal Replacement Therapy; HS, Hepatorenal syndrome; HE,Hepatic encephalopathy; PH,Portal hypertension; SBP,Spontaneous bacterial peritonitis.


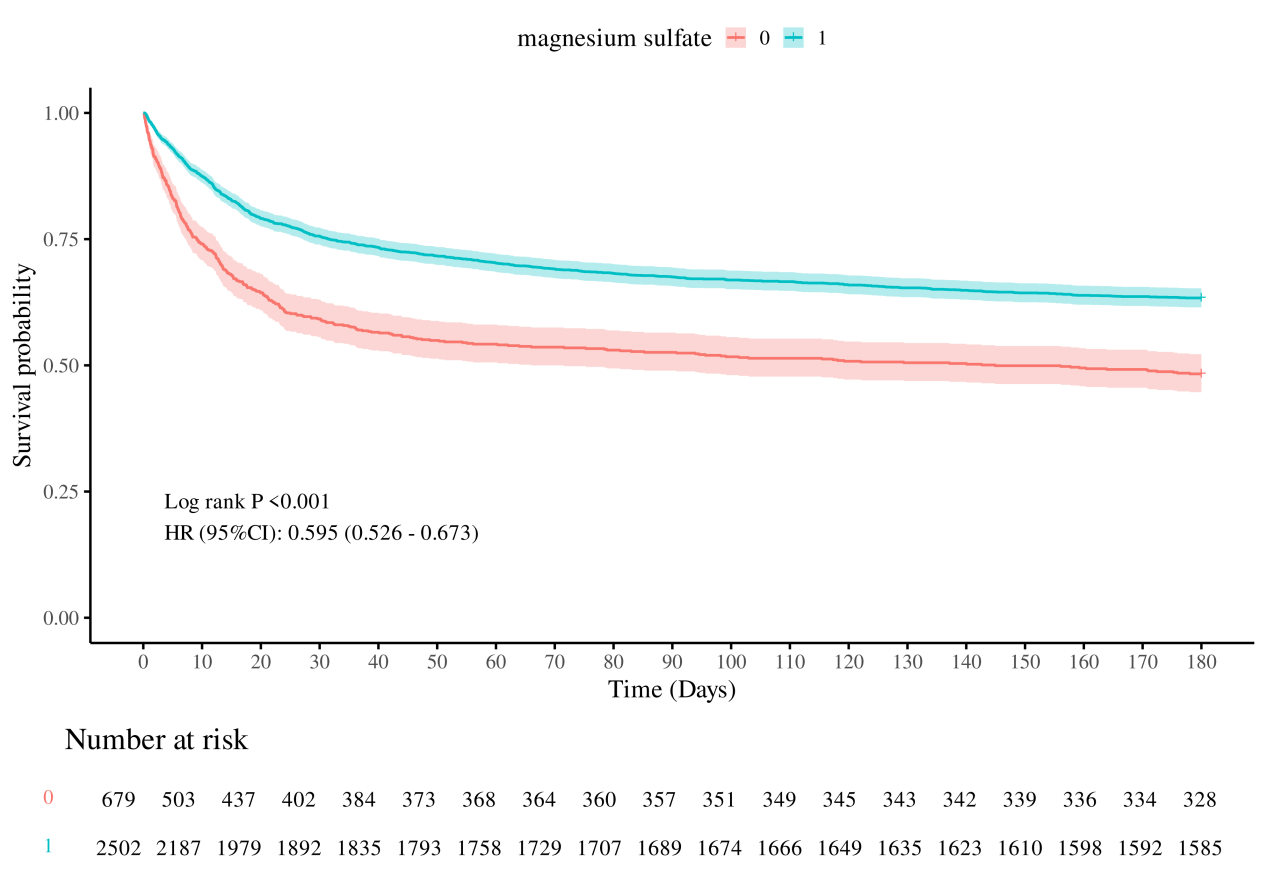


**Supplementary material S12** Kaplan-Meier survival curves of 180-day all-cause mortality. 0:Magnesium sulfate not used 1:Magnesium sulfate used.

(A)


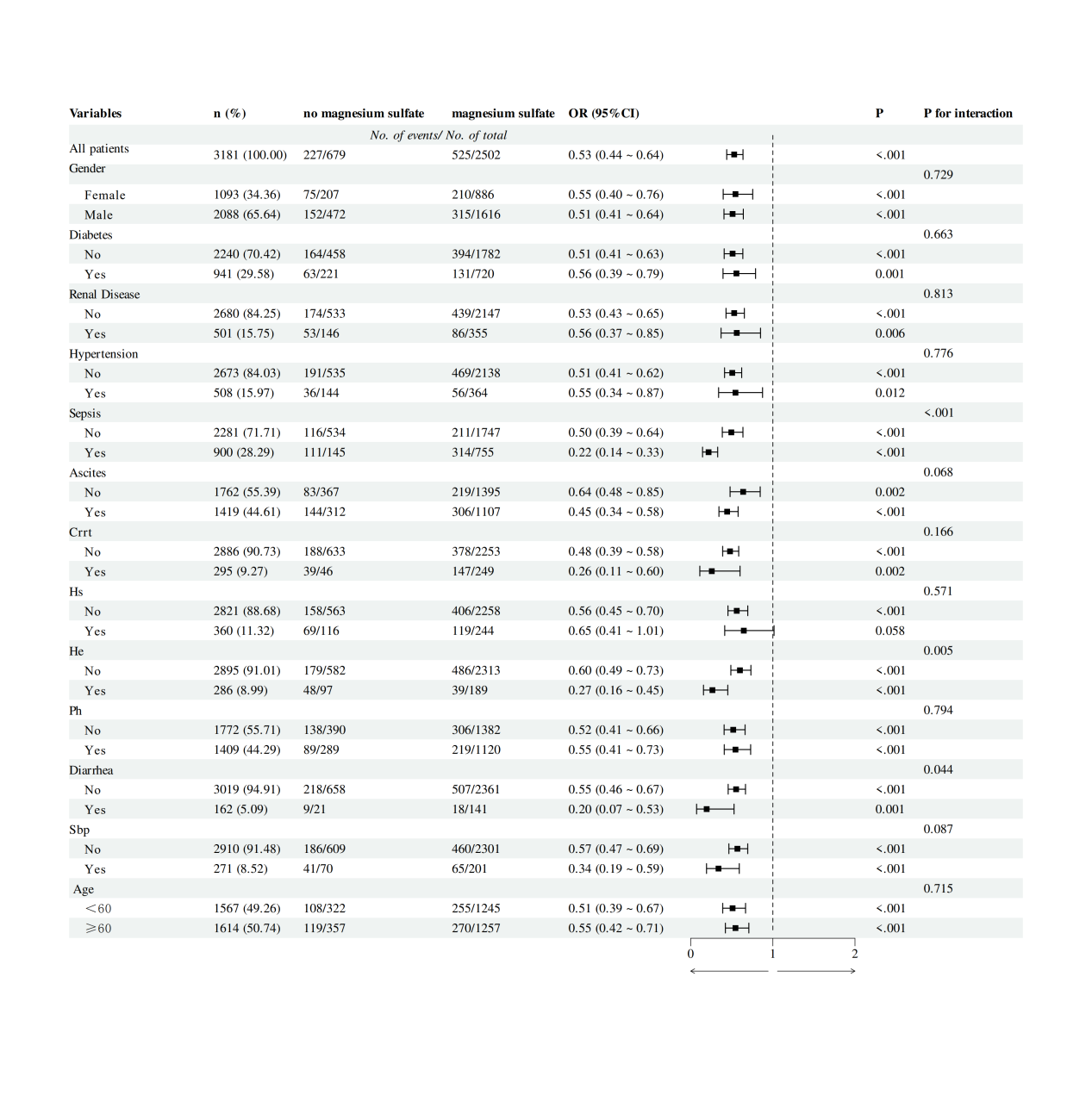


(B)


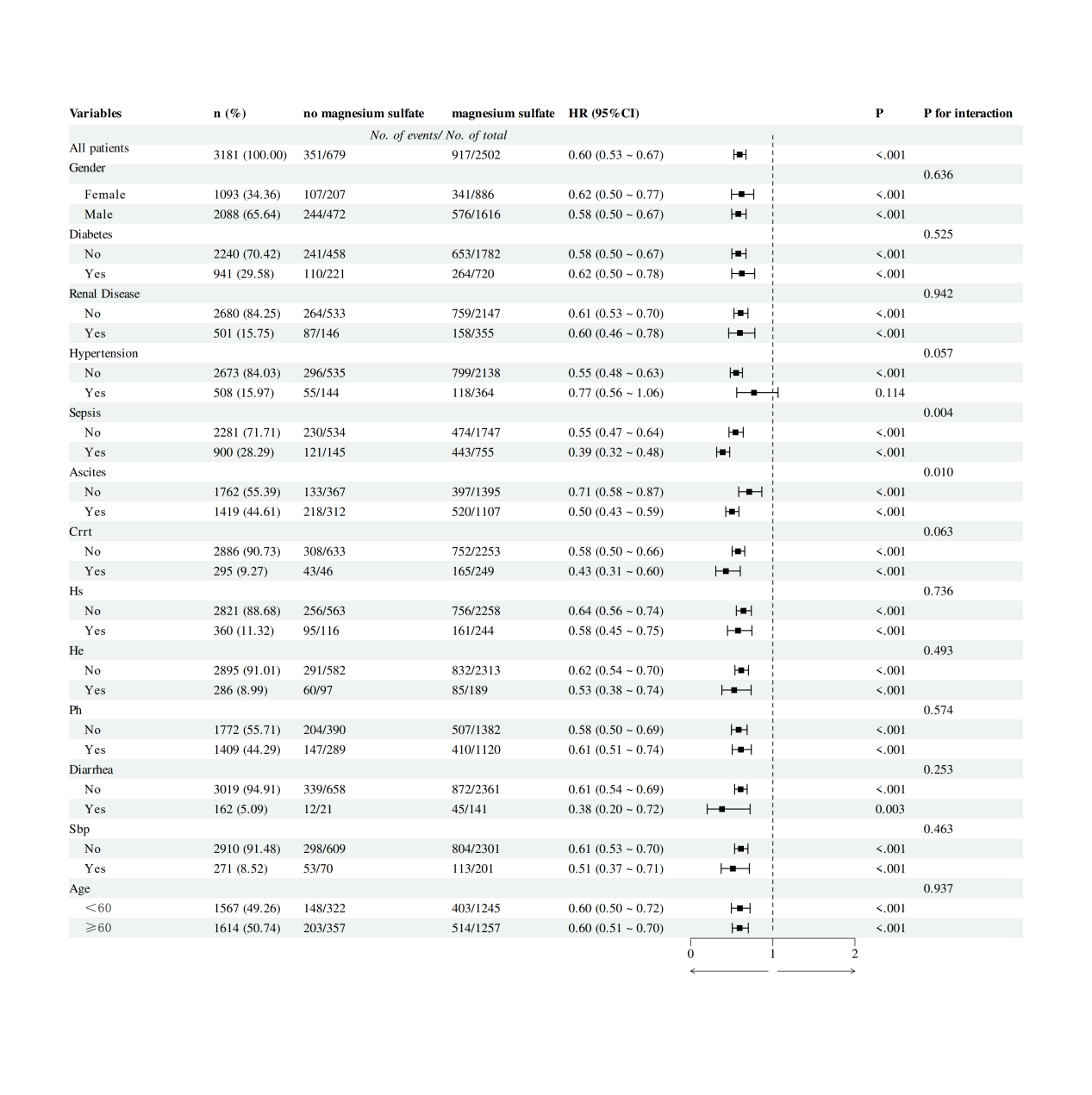


**Supplementary material S13** Subgroup analysis of the association between magnesium sulfate use and outcomes in critically ill patients with cirrhosis.

(A)In-hospital mortality logistics regression subgroup analysis forest plot.

(B)180-day mortality Cox regression subgroup analysis forest plot.

Abbreviation:CRRT, Continuous Renal Replacement Therapy; HS, Hepatorenal syndrome; HE,Hepatic encephalopathy; PH,Portal hypertension; SBP,Spontaneous bacterial peritonitis.
